# Changes in US Primary Care Access and Capabilities During the COVID-19 Pandemic

**DOI:** 10.1001/jamahealthforum.2024.5237

**Published:** 2025-02-07

**Authors:** Matthew Mackwood, Elliott Fisher, Rachel O. Schmidt, Ching-Wen W. Yang, A. James O’Malley, Hector P. Rodriguez, Stephen Shortell, Ellesse-Roselee L. Akré, Karen E. Schifferdecker

**Affiliations:** 1 Department of Community and Family Medicine, Geisel School of Medicine at Dartmouth, Lebanon, New Hampshire; 2 The Dartmouth Institute for Health Policy and Clinical Practice, Geisel School of Medicine at Dartmouth, Lebanon, New Hampshire; 3 Department of Medicine, Geisel School of Medicine at Dartmouth, Lebanon, New Hampshire; 4 Department of Biomedical Data Science, Geisel School of Medicine at Dartmouth, Lebanon, New Hampshire; 5 School of Public Health, University of California, Berkeley; 6 Department of Health Policy and Management, Johns Hopkins University Bloomberg School of Public Health, Baltimore, Maryland

## Abstract

**Importance:**

Many of the capabilities needed to deliver accessible, high-quality primary care have been defined, but little is known about how their implementation has changed in US practices over the course of the COVID-19 pandemic or about the factors associated with greater capabilities.

**Objective:**

To describe US primary care practices’ accessibility and capabilities and examine recent changes.

**Design, Setting, and Participants:**

This was a retrospective cohort study across 2 surveys, in 2017 to 2018 and 2022 to 2023, among a national sample of primary care practice leaders in the US. Data were analyzed from January 2023 to September 2024.

**Exposures:**

Degree of integrated practice ownership and accountable care organization (ACO) participation.

**Main Outcomes and Measures:**

Differences by practice ownership and ACO participation, and changes over time in access to care and care delivery capabilities. These were measured by composite scores of responses standardized to a scale of 0 to 100.

**Results:**

This analysis included 710 practices, of which 234 were independently owned, 105 were physician group owned, and 321 were hospital/health system owned in 2017 to 2018, and 68 practices reported no ACO participation, 107 joined between surveys, and 486 otherwise participated in ACOs. Access to care (measured as extended weekday or weekend hours) was reported to decline from the first survey in 2017 to 2018 to the second in 2022 to 2023. Hospital/health system practices and ACO participants had higher rates of extended weekday hours than their comparators in 2022 to 2023. Average capability scores increased from 51 to 54 (increase of 4 points [95% CI, 1-6 points]). There was wide variation in scores within all ownership and ACO participant or nonparticipant groups. Capability scores were higher on average for more integrated practices (for physician groups compared to independent practices, 12 points [95% CI, 5-19 points] in 2017-2018 and 12 points [95% CI, 7-16 points] in 2022-2023) and for ACO participants compared to nonparticipants (13-point difference [6 to 20] in 2017-2018 and 12-point difference [6 to 18] in 2022-2023).

**Conclusions and Relevance:**

In this cohort study, over the time period including the COVID-19 pandemic, primary care practices reported a decline in access to care, while average practice capabilities improved. Integrated practice ownership and ACO participation were both associated with better access and capability scores, suggesting that value-based payment and integrated care delivery support the development of higher-quality primary care. Variations across practices point to large opportunities for improvement overall and underscore the importance of incentives and structures as levers to improve primary care delivery.

## Key Points

**Question:**

How did the capabilities and accessibility of US primary care practices change in recent years?

**Findings:**

In this cohort study across 710 primary care practices that completed surveys in 2017 to 2018 and 2022 to 2023, access to primary care was reported to decline, yet modest improvements in capabilities were reported. Practices participating in accountable care organizations and those with more integrated ownership reported higher scores on average, though scores varied substantially within groups.

**Meaning:**

Further attention to primary care’s accessibility and capabilities is necessary to inform ongoing efforts to enhance and expand high-quality primary care in the US.

## Teaser

This cohort study describes US primary care practices’ accessibility and capabilities and examines recent changes.

## Introduction

Primary care plays an essential role in the prevention and management of most chronic conditions affecting the US population.^1^ The capabilities for providing high-quality primary care have been increasingly well defined over time^2^ and were highlighted in a 2021 report by the National Academies of Science, Engineering, and Medicine (NASEM).^3^ These capabilities include effective integration of screening for clinical conditions^4^ and social needs,^5^ behavioral health integration,^6^ the use of process-improvement systems,^7^ team-based care models for patients with complex needs and a high level of need,^8^ and an emphasis on population health through electronic health record (EHR) integration to support panel-based registries for preventive and chronic disease management.^9,10^ Recognizing the importance of timely access to care, many primary care practices have used advanced access models, which prioritize same-day visits, and offered extended weekday or weekend clinic hours, which can reduce wait times and decrease urgent and emergency care use.^11,12,13^

Despite what is known about providing high-quality primary care, the extent to which these capabilities have changed over time is largely unknown. Concern about the impact of COVID-19 on the primary care workforce has been well documented,^14^ but less is known about how specific capabilities and access to care have changed over the course of the pandemic, particularly in light of longer-term trends affecting primary care: consolidation of ownership of primary care practices by hospitals and health systems has increased,^1,15^ as has participation in alternative payment models (especially in accountable care organizations [ACOs]^16^). However, the degree to which these trends are associated with changes in capabilities and accessibility is unknown.^17^

To better understand the current state of primary care in the US amid these trends, we compare findings from the 2017 to 2018 and 2022 to 2023 surveys of a national sample of physician practices, focusing on care accessibility, implementation of evidence-informed care delivery processes, and the association of practice ownership and ACO participation with any observed differences.

## Methods

### Study Design

We carried out a longitudinal cohort study of US primary care practices as a component of the National Survey of Healthcare Organizations and Systems, extending an earlier series of national surveys of primary care practices.^18,19^ The initial (2017-2018) and follow-up (2022-2023) surveys were developed through an iterative process that included extensive literature reviews, engaging national experts and practice leaders in the nomination of potentially important domains, identification of previously validated survey items, and the development and testing of new items and scales. The study was approved by the Dartmouth College Committee for the Protection of Human Subjects. Since primary care practices were the unit of analysis, informed consent was not required for this study. This study is reported in accordance with the Strengthening the Reporting of Observational Studies in Epidemiology (STROBE) reporting guidelines.^20^

### Data Sources and Sampling Strategy

Sample frames for the surveys were drawn from IQVIA’s OneKey database,^21^ which is a roster of clinician practices, the prescribers located at each site, and the organizations that own each practice. These data have been used previously for national reporting on health system ownership and evaluations of federal primary care payment reform initiatives.^22,23,24,25^ The initial survey randomly sampled practices with at least 3 adult primary care clinicians (physicians or advanced practice professionals, including physician assistants and nurse practitioners with specialties of family practice, general practice, internal medicine, or geriatric medicine), stratified by the degree of integrated ownership: independent practices, practices within medical groups (no hospital or system ownership), or integrated delivery systems (practices owned by a hospital or health system). The initial survey methods are discussed in detail elsewhere.^26^ All initial survey respondents (response rate, 43%) were included in the follow-up survey; of 1959 practices surveyed in the follow-up, 722 (37%) responded and 710 were included in this analysis after exclusions. Additional details, including a respondent flowchart and sample frame characteristics, are provided in eFigure 1 and eTable 1 in Supplement 1.

### Survey Development, Contents, and Administration

The primary outcomes are the degree to which practices reported the adoption of care delivery processes (referred to hereafter as capabilities) and payment reforms hypothesized or shown to be associated with better performance. Details about the initial survey items and scales were described previously^17^ and are summarized in the eMethods in Supplement 1. To characterize access to care, we report on rates of extended weekday (before 8 am or after 5 pm) or weekend clinic hours (both explicitly excluding urgent care) and the use of advanced access scheduling. Scales based on the items included in both surveys were updated, calculated as arithmetic averages of reported standardized items to allow for item nonresponse, and tested for reliability. The 9 individual capability scores are described in eTable 2 in Supplement 1, including the wording of all questions included in this analysis. We also generated an average capability score, using all items in the individual capability scores. Both surveys underwent several rounds of cognitive interviewing with practice leaders. Ownership and clinician counts for each practice and time period were ascertained from the OneKey database (from 2017 and 2022, respectively). Rurality was defined using rural-urban commuting area codes based on practice business address.

### Statistical Analysis

We estimated linear regression models to examine differences in levels and changes over time in capabilities and access. We used univariate ordinary least squares regression to estimate associations between groups of interest and capability scores or accessibility within each period; χ^2^ tests were used to compare categorical factors. To estimate the association of changes over time in capabilities and access with ACO participation and ownership, we included indicators of each as possible predictors in multivariable regression models. Each possible predictor was interacted with a binary indicator of the 2022 to 2023 survey to allow associations to be estimated while accounting for time-varying changes and levels. Data were analyzed in Stata, version 17 (StataCorp), and data were analyzed from January 2023 to September 2024.

Weights were used in all analyses to account for nonresponse probabilities and the complex survey design that sampled practices within larger organizations that owned the practice (additional details are provided in the eMethods in Supplement 1). We used robust standard error estimations for 95% CIs and tests of significance to obtain appropriately calibrated statistical tests. Average marginal effects were used to report adjusted estimates and 95% CIs. All tests were 2-sided, with *P* < .05 indicating statistical significance.

### Sensitivity Analysis

To further examine the associations between integrated ownership and ACO payment participation on average capability score changes, we ran a multilevel mixed-effects model with the same indicators as in the aforementioned multivariable model. This model included random effects to account for clustering by system.

## Results

### Practice Characteristics

Practice characteristics for both surveys are compared in Table 1 (characteristics stratified by ACO participation and ownership are provided in eTables 3 and 4 in Supplement 1). Independent ownership decreased from 37% to 31%, hospital or health system ownership grew from 40% to 49%, and physician group ownership decreased from 13% to 10%. No practices left ownership by a hospital/health system or medical group. There was no change in physician employment per practice, while advanced practice professional employment grew. While ACO participation increased from 1.2 to 1.6 contracts of different payer types per practice on average, 43% of practices reported contracts with 1 or no types of payers in 2022 to 2023.

**Table 1.** Practice Characteristics by Survey Year

Characteristic	Practices, weighted % (unweighted count)			*P* value for difference between years^a^
	Total (N = 710)	Survey year		
		2017-2018	2022-2023	
Ownership				
Independent	NA	37 (234)	31 (193)	<.001
Physician group	NA	13 (105)	10 (85)	
Hospital or health system	NA	40 (321)	49 (380)	
FQHC (regardless of ownership)	NA	10 (50)	10 (52)	
ACO participation				
No. of ACOs by payer type, mean (SD)^b^	NA	1.2 (0.1)	1.6 (0.1)	<.001
No. of ACO payer types				
0	NA	36 (193)	29 (153)	<.001
1	NA	24 (149)	14 (97)	
2	NA	20 (132)	21 (150)	
3	NA	20 (146)	36 (246)	
No. of physicians in practice				
0-4	NA	47 (281)	48 (286)	.51
5-9	NA	28 (227)	27 (217)	
10-19	NA	14 (109)	14 (105)	
≥20	NA	10 (93)	11 (96)	
No. of advanced practice professionals in practice^c^				
0	NA	20 (131)	18 (126)	.01
1 or 2	NA	36 (275)	32 (238)	
3 or 4	NA	22 (149)	23 (158)	
5-10	NA	14 (98)	18 (113)	
≥10	NA	8 (57)	9 (69)	
Ratio of advanced practice professionals to physicians				
<0.5	NA	59 (469)	52 (393)	<.001
0.5 to <1	NA	21 (138)	23 (148)	
1 to <2	NA	17 (90)	15 (123)	
≥2	NA	3 (13)	11 (37)	
Changes in ownership				
Group to hospital/health system	5 (39)	NA	NA	NA
Hospital to system	7 (51)	NA	NA	NA
Independent to FQHC	0.1 (2)	NA	NA	NA
Independent to group	2 (19)	NA	NA	NA
Independent to hospital/health system	3 (20)	NA	NA	NA
No change	82 (579)	NA	NA	NA
Changes in ACO participation				
No ACO participation (at either survey)	15 (68)	NA	NA	NA
Joined ACO between surveys	16 (107)	NA	NA	NA
Other ACO participation	69 (486)	NA	NA	NA
Practice in a rural location^d^	7 (60)	NA	NA	NA
US Census region				
New England	8 (61)	NA	NA	NA
Middle Atlantic	14 (84)	NA	NA	NA
East North Central	12 (108)	NA	NA	NA
West North Central	11 (81)	NA	NA	NA
South Atlantic	17 (120)	NA	NA	NA
East South Central	4 (23)	NA	NA	NA
West South Central	7 (49)	NA	NA	NA
Mountain	9 (70)	NA	NA	NA
Pacific	18 (114)	NA	NA	NA

Abbreviations: ACO, accountable care organization; FQHC, federally qualified health center; NA, not applicable.

^a^ χ^2^ Test.

^b^ Commercial, Medicare, or Medicaid.

^c^ Includes physician assistants and nurse practitioners with specialties of family practice, general practice, internal medicine, or geriatric medicine.

^d^ Determined by rural-urban commuting area classification for the practice’s business address.

### General Trends in Access to Care and Practice Capabilities

Table 2 describes changes in access to care and capability scores over time; eTable 5 in Supplement 1 presents medians and IQRs for select items. Accessibility declined, highlighted by a substantial decrease in practices offering weekend hours (from 44% to 26% [−18 percentage points; 95% CI, −24 to −12 percentage points]) and a decline in advanced access scheduling, with only 26% of practices routinely using this approach in 2022 to 2023 compared to 60% in 2017 to 2018 (−34 percentage points; 95% CI, − 41 to −27 percentage points).

**Table 2.** Changes in Primary Care Practice Capabilities and Accessibility Between 2017-2018 and 2022-2023

Domain	Survey years		Difference (95% CI)^a^
	2017-2018	2022-2023	
Accessibility, weighted % of respondents			
Offer extended hours^b^	61	51	−10 (−15 to −4)
Offer weekend visits	44	26	−18 (−24 to −12)
Use advanced access scheduling (most or all health care professionals)	60	26	−34 (−41 to −27)
Practice capability composite scores, mean on 100-point scale			
Average capability score (excluding access)	51	54	4 (1 to 6)
Behavioral health integration	41	41	0.5 (−5 to 6)
Motivational interviewing training	42	45	3 (−3 to 9)
Depression care processes	67	72	6 (2 to 9)
Screening for social needs	37	43	7 (1 to 12)
Care of patients with complex/high needs	46	61	15 (12 to 19)
Electronic health record integration	59	67	9 (5 to 12)
Patient-reported outcome measures	63	70	7 (2 to 12)
Screening for clinical conditions	75	76	0.8 (−4 to 55)
Physician and clinic improvement processes	44	41	−3 (−7 to 0.2)
Chronic disease management processes	52	53	1 (−4 to 7)

^a^ Values may differ due to rounding. Accessibility differences are reported in percentage points.

^b^ Visits before 8 am or after 5 pm on weekdays.

Average capability scores were reported to improve from 51 to 54 on a 100-point scale (increase of 4 points; 95% CI, 1-6 points). Among individual capabilities, the largest improvement was in the care of patients with complex/high needs, where the score improved by 15 points (95% CI, 12-19 points), from 46 to 61. Meaningful improvement also occurred in EHR integration and depression care processes (59 to 67 [increase of 9 points; 95% CI, 5-12 points] and 67 to 72 [increase of 6 points; 95% CI, 2-9 points], respectively), with fewer improvements in the use of patient-reported outcome measures and screening for social needs (63 to 70 [increase of 7 points; 95% CI, 2-12 points] and 37 to 43 [increase of 7 points; 95% CI, 1-12 points], respectively). There was not a meaningful change in all other capabilities over time, with the exception of a decline in use of physician and clinic improvement processes (44 to 41 [−3 points; 95% CI, −7 to 0.2 points]).

### Variation in Access and Capabilities by Ownership and ACO Status

The Figure and eTable 9 in Supplement 1 present the distribution of average capability scores over time, both in total and stratified by ACO payment participation and practice ownership. Average capabilities improved overall, while variation narrowed slightly across surveys. Practices that joined an ACO between surveys and those owned by physician groups and hospital/health systems improved scores among practices with lower scores.

**Figure.**
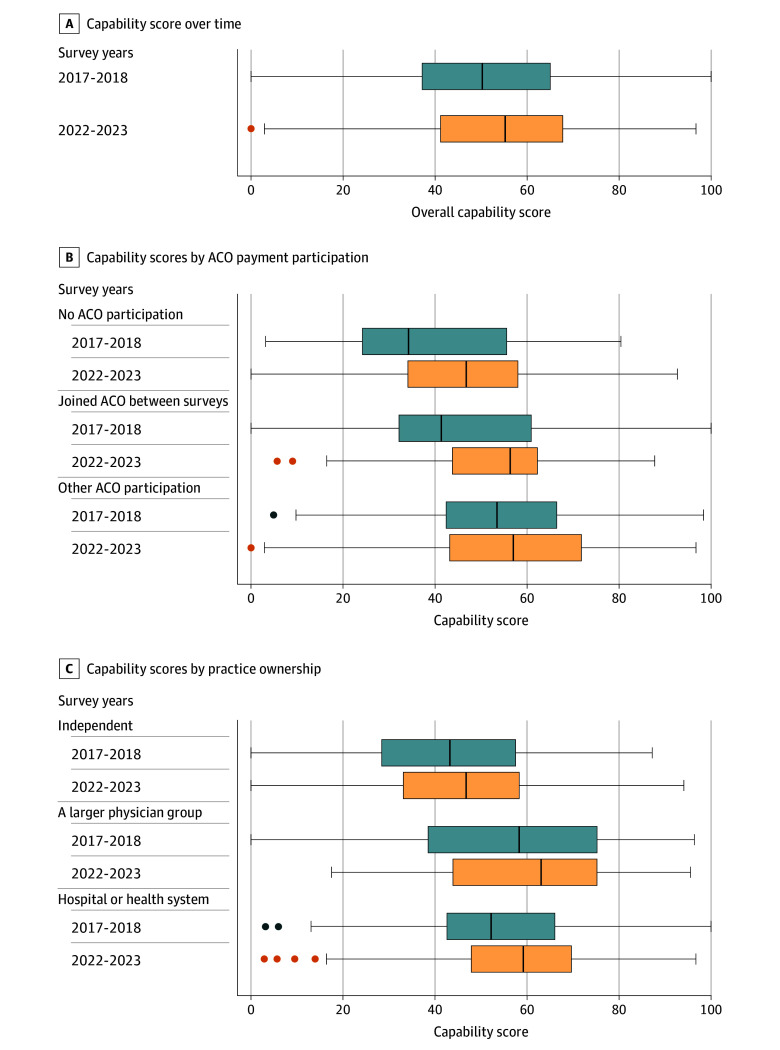
Average Practice-Level Composite Capability Score, Stratified by Accountable Care Organization (ACO) Participation and Practice Ownership Central lines denote the median, boxes represent the IQR, whiskers display 1.5 times the IQR bounded by maximum or minimum as applicable, and dots represent outliers beyond 1.5 times the IQR. See eTable 9 in Supplement 1 for a distribution of average capability scores over time, both in total and stratified by ACO payment participation and practice ownership.

Tables 3 and 4 present levels and changes over time in select practice capabilities and accessibility by ACO payment participation and ownership. A full listing of levels, including tests of statistical significance between groups, is provided in eTables 6 and 7 in Supplement 1. Accessibility declined across all strata, though ACO participants reported higher rates of extended-hours access compared to nonparticipants in 2022 to 2023 (55% vs 34% [difference, 22 percentage points; 95% CI, 5-39 percentage points]), as did hospital/health system–owned practices compared to independent practices (54% vs 38% [difference, 16 percentage points; 95% CI, 4-28 percentage points]).

**Table 3.** Changes in Primary Care Accessibility and Select Practice Capabilities Between 2017 and 2022, Stratified by Accountable Care Organization (ACO) Participation

Domain	No ACO participation (n = 68)			Joined ACO between surveys (n = 107)			All other ACO participation (n = 486)		
	Survey years		Adjusted difference (95% CI)^a^	Survey years		Adjusted difference (95% CI)^a^	Survey years		Adjusted difference (95% CI)^a^
	2017-2018	2022-2023		2017-2018	2022-2023		2017-2018	2022-2023	
Accessibility, weighted % of respondents									
Offer extended hours^b^	38	34	4 (−22 to 13)	61	50	−10 (−25 to 5)	65	55	−11 (−17 to −4)
Offer weekend visits	18	20	5 (−20 to 30)	57	26	−31 (−44 to −17)	47	27	−18 (−24 to −13)
Select practice capability composite scores, mean on 100-point scale									
Average capability score (excluding access)	41	45	4 (−3 to 11)	47	53	6 (−1 to 12)	54	57	3 (0.4 to 6)
Behavioral health integration	41	23	−22 (−41 to 2)	36	40	3 (−7 to 14)	40	45	2 (−4 to 9)
Depression care processes	59	61	3 (−8 to 14)	62	74	13 (5 to 21)	70	75	4 (0.9 to 8)
Care of patients with complex/high needs	34	64	29 (21 to 36)	46	55	9 (−2 to 19)	49	63	14 (9 to 18)
Chronic disease management processes	36	40	4 (−16 to 24)	40	54	14 (0.5 to 27)	59	56	−4 (−10 to 2)

^a^ Values may differ due to rounding. Adjusted for ACO participation and ownership. Accessibility adjusted differences are reported in percentage points.

^b^ Visits before 8 am or after 5 pm on weekdays.

**Table 4.** Changes in Primary Care Accessibility and Select Practice Capabilities Between 2017 and 2022, Stratified by Ownership

Domain	Independently owned			Physician group owned			Hospital/health system owned		
	2017-2018 Survey (n = 234)	2022-2023 Survey (n = 193)	Adjusted difference (95% CI)^a^	2017-2018 Survey (n = 105)	2022-2023 Survey (n = 85)	Adjusted difference (95% CI)^a^	2017-2018 Survey (n = 321)	2022-2023 Survey (n = 380)	Adjusted difference (95% CI)^a^
Accessibility, weighted % of respondents									
Offer extended hours^b^	53	38	−15 (−26 to −3)	69	53	−15 (−31 to 1)	60	54	−5 (−12 to 2)
Offer weekend visits	49	30	−20 (−32 to −7)	49	34	−12 (−27 to 4)	36	20	−15 (−20 to −10)
Select practice capability composite scores, mean on 100-point scale									
Average capability score (excluding access)	45	47	2 (−3 to 7)	56	59	3 (−3 to 9)	53	58	5 (2 to 8)
Behavioral health integration	21	19	−0.4 (−10 to 9)	33	26	−10 (−25 to 6)	48	48	−0.4 (−9 to 8)
Depression care processes	66	68	2 (−4 to 9)	72	76	4 (−3 to 10)	65	74	10 (5 to 14)
Care of patients with complex/high needs	41	62	20 (13 to 27)	55	66	14 (5 to 22)	47	60	13 (9 to 17)
Chronic disease management processes	45	42	−3 (−13 to 9)	62	63	2 (−11 to 15)	56	61	5 (−2 to 11)

^a^ Values may differ due to rounding. Adjusted for accountable care organization participation and ownership. Accessibility adjusted differences are reported in percentage points.

^b^ Visits before 8 am or after 5 pm on weekdays.

When stratifying by ACO participation, capability scores in both time periods were highest for those in ACOs, lowest for nonparticipants, and intermediate for those who joined between surveys. Differences were largest when comparing ACO participants to nonparticipants (13-point difference [6 to 20] in 2017-2018 and 12-point difference [6 to 18] in 2022-2023). All groups reported statistically similar changes over time in average capability score (there were no statistically significant difference in differences), but there were different magnitudes of change in a few individual capabilities. Each group in the ACO participation strata improved on the care of patients with complex/high needs, with nonparticipants experiencing higher change to converge from a lower baseline (Table 3). ACO nonparticipants reported a considerable decline in behavioral health integration, while the other groups were unchanged. While not statistically distinct from other groups, those who joined ACOs made substantial increases in chronic disease management processes (14 points; 95% CI, 0.5-27 points) and depression care processes (13 points; 95% CI, 5-21 points).

Across ownership strata, average capability scores were higher for practices owned by physician groups and hospitals/health systems compared to independently owned practices (for physician groups, difference of 12 points [95% CI, 5-19 points] in 2017-2018 and 12 points [95% CI, 7-16 points] in 2022-2023). Similar trends held across individual capabilities, most notably in EHR integration, physician and clinic improvement processes, and chronic disease management processes (eTable 7 in Supplement 1). There were no statistically significant differences when comparing changes in capability scores across ownership strata. Change in ownership between surveys was not associated with differential changes in practice capabilities in multivariable modeling.

### Sensitivity Analysis

Similar to the primary analysis, multivariable, multilevel mixed-effects modeling of changes in average capability scores improved over time for ACO participants and hospital/health system–owned practices (4 points [95% CI, −1 to 9 points] and 5 points [95% CI, 2-8 points], respectively; eTable 8 in Supplement 1). Modeling of differences in average capability score by ACO participation and ownership type was different for integrated ownership and ACO participation, though the estimate for ACO participation was 5 points higher (95% CI, −0.2 to 9 points) than nonparticipation.

## Discussion

This longitudinal study of a national sample of primary care practices is, to our knowledge, the first to examine trends in accessibility, practice capabilities, ownership, and payment model participation between the pre–COVID-19 pandemic period of 2017 to 2018 and the 2022 to 2023 period of transition from pandemic to endemicity.^27^ Average capability scores increased by 4 points on a 100-point scale. A 1-point change in the average capability score corresponds to a 2-point shift in percentile ranking, meaning that the average practice in 2022 to 2023 improved its rank by 8 percentage points on the 2017 to 2018 distribution. On average, practices participating in ACO payment models and those owned by physician groups, hospitals, or health systems reported greater accessibility and practice capabilities. The magnitude of the difference in average capability score within each survey for these groups is equivalent to a about a 25-point percentile shift. Changes in capabilities over time were similar between groups, with the exception of better preservation of behavioral health integration among those joining or already participating in ACO payment models. While we believe that the magnitude of the differences among groups and some of the changes over time are important, we recognize that others may choose a different threshold. To help visualize the magnitude of changes in capabilities, readers can examine eFigures 2 through 4 in Supplement 1, which show score distributions across ACO payment and ownership strata.

The COVID-19 pandemic likely exacerbated the decline in accessibility of primary care, perhaps due to ongoing issues with staffing and burnout.^28,29^ As the 2024 primary care scorecard “No One Can See You Now” reports, prepandemic trends in the workforce have remained inadequate to the needs of the US population.^30^ The decline in the use of same-day advanced access scheduling could also reflect restructured scheduling protocols, which included previsit COVID-19 testing to protect office staff and other patients in many practices.

The greatest change was seen in the adoption of care processes for patients with complex/high need, where the average increase in score equated to an approximate 25–percentile point improvement on the 2017 to 2018 distribution for this capability. These care processes incorporate clinical care teams and directly reflect activities that are reimbursed by the Centers for Medicare & Medicaid Services’ fee schedule for transitional care (introduced in 2014) and chronic care management (introduced in 2015 and augmented in 2020),^31^ which is a likely factor in the observed improvement. Other capabilities were higher under ACO payment models or hospital or health system ownership; such practices may be more likely to have the resources needed to implement evidence-based processes and to require and support integration with a larger system’s EHRs.

The present findings show gains in ACO participation by practices and evidence that both private and public sector payers are now participating. However, the most recent American Medical Association data show that 86% of practices are still receiving at least some fee-for-service revenue, 69% of overall physician practice revenue flows through fee-for-service (a share that has been steady across the past decade), and only 25% and 22% of practices received any capitation or shared savings payments, respectively.^32^ Given these low proportions of value-based reimbursement, the relative magnitude of differences in reported capabilities between nonparticipants and participants in ACO payment models is striking and suggests that continued exploration of how to accelerate a transition to all-payer ACO models is warranted.

These findings are relevant to the persistent concerns about the challenges facing primary care highlighted in the NASEM report.^3^ Despite the growing evidence base supporting the benefits of capabilities such as those in this study,^2,3,4,5,6,7,8,9,10,11,12,13^ these findings align with others in showing that the US is far from achieving their universal implementation.^3^ The wide range of variation suggests that additional research into effective oversight and evaluation of primary care on an ongoing basis will be important to help accelerate improvement.^33^ At the same time, this study finds that with the notable exception of access to care, most capabilities improved over the course of the pandemic. In our analysis, ACOs and integrated ownership were both associated with improving capabilities over time and may both help to strengthen primary care delivery, but they have different implications: integrated ownership may be associated with greater consolidation within a given market, reducing competition, which could lead to both higher prices and weaker incentives to improve care.^34^ At the same time, the administrative burden associated with the quality incentives in value-based payment plans comes with costs,^35^ contributes to clinician burnout,^36^ and results in payments to the parent organizations that are not necessarily passed through to support primary care delivery.^37^ These findings are, therefore, aligned with the NASEM committee’s recommendation to increase investment in primary care through payment reform^37^ and to ensure that payment for care quality is not only appropriately incentivized, but also effectively allocated.

### Limitations

This study has important limitations. Without further work to validate actual capabilities against what is reported, survey responses may be subject to favorability bias. Response rates for 2022 to 2023 were lower than for 2017 to 2018. The analyses of both surveys were weighted to account for nonresponse and to best reflect the national sample frame, with eTable 1 in Supplement 1 showing that respondents and nonrespondents were similar on most of the observable measures available from the OneKey data. Unobserved differences between respondents and nonrespondents may limit external validity for comparisons across groups. The findings may not generalize to practices with 2 or fewer physicians, which this sample frame excluded. However, while such practices accounted for nearly 60% of primary care practices in 2021, they only represented approximately 15% of practicing primary care physicians.^22^ The surveyed measures of access do not capture nonvisit modes (such as clinician telephone calls or patient messaging) or overall volumes of care, and the 2022 to 2023 survey’s results need correlating to larger trends in utilization. Studies of the 2017 to 2018 survey demonstrated that more robust capabilities were linked to differential utilization and lower costs of care^38,39^ and have found limited differences in outcomes in breast cancer screening^40^ and musculoskeletal conditions.^41^ Finally, while we are not able to definitively demonstrate causal links among the associations observed in this study, nor link them to changes in patient outcomes, this study finds meaningful changes over time in a national sample of practices, and these findings can help generate hypotheses that can be tested in future work to enhance understanding of how to improve primary care delivery.

## Conclusions

In this cohort study of primary care practices surveyed in 2017 to 2018 and 2022 to 2023, we found large declines in accessibility and modest improvements in capabilities. Substantial variation in capabilities and, thus, room for improvement is notable across all types of practices. Participation in ACO payment models and ownership by hospitals or health systems were associated with better preserved access and higher practice capabilities. While this analysis cannot determine the extent to which the COVID-19 pandemic itself or other factors underlie these associations, the findings suggest that payment reform and primary care ownership are highly relevant to enhancing the capabilities essential to deliver high-quality, accessible primary care.

## Notes

Supplement 1.
eMethods
**eFigure 1.** Respondent Flow Chart **eFigure 2.** Average & Select Capability Score Distributions (Median & IQR), with Stratified Adjusted Means & 95% CI **eFigure 3.** Additional Capability Score Distributions (Median & IQR), with Stratified Adjusted Means & 95% CI **eFigure 4.** Capability Score Distributions For Binary Items (Overall Mean & 95% CI) with Stratified Adjusted Means & 95% CI **eTable 1.** Comparison of Sample Frame and Study Population at Time of Initial Assignment (2015) **eTable 2. **Composite Capability Score Items **eTable 3.** Practice Characteristics by Survey Year Stratified by Accountable Care Organization Participation **eTable 4.** Practice Characteristics by Survey Year, Stratified by Ownership **eTable 5.** Unweighted Median and Interquartile Ranges for Select Capability Scores **eTable 6.** Detailed Comparisons of Primary Care Practice Capabilities, Stratified by ACO Participation **eTable 7.** Detailed Comparisons of Primary Care Practice Capabilities, Stratified by Ownership **eTable 8.** Comparison of Average Capability Score by ACO Participation and Ownership from Multi-level, Mixed-effects Model **eTable 9.** Distribution of Average Capability Scores Over Time, in Total and Stratified by ACO Payment Participation and Practice Ownership

Supplement 2. Data Sharing Statement
