# Supplementary material for: Changes in US Primary Care Access and Capabilities During the COVID-19 Pandemic
Source: JAMA Health Forum. 2025 Feb 7;6(2):e245237. doi: 10.1001/jamahealthforum.2024.5237 (PMC11806387; doi:10.1001/jamahealthforum.2024.5237)
Supplement: Supplement 1. — eMethods eFigure 1. Respondent Flow Chart eFigure 2. Average & Select Capability Score Distributions (Median & IQR), with Stratified Adjusted Means & 95% CI eFigure 3. Additional Capability Score Distributions (Median & IQR), with Stratified Adjusted Means & 95% CI eFigure 4. Capability Score Distributions For Binary Items (Overall Mean & 95% CI) with Stratified Adjusted Means & 95% CI eTable 1. Comparison of Sample Frame and Study Population at Time of Initial Assignment (2015) eTable 2. Composite Capability Score Items eTable 3. Practice Characteristics by Survey Year Stratified by Accountable Care Organization Participation eTable 4. Practice Characteristics by Survey Year, Stratified by Ownership eTable 5. Unweighted Median and Interquartile Ranges for Select Capability Scores eTable 6. Detailed Comparisons of Primary Care Practice Capabilities, Stratified by ACO Participation eTable 7. Detailed Comparisons of Primary Care Practice Capabilities, Stratified by Ownership eTable 8. Comparison of Average Capability Score by ACO Participation and Ownership from Multi-level, Mixed-effects Model eTable 9. Distribution of Average Capability Scores Over Time, in Total and Stratified by ACO Payment Participation and Practice Ownership [file jamahealthforum-e245237-s001.pdf]

Supplemental Online Content

Mackwood M, Fisher E, Schmidt RO, et al. Changes in US primary care access and capabilities during the COVID-19 pandemic. *JAMA Health Forum*. 2025;6(2):e245237. doi:10.1001/jamahealthforum.2024.5237

eMethods

eFigure 1. Respondent Flow Chart

eFigure 2. Average & Select Capability Score Distributions (Median & IQR), with Stratified Adjusted Means & 95% CI

eFigure 3. Additional Capability Score Distributions (Median & IQR), with Stratified Adjusted Means & 95% CI

eFigure 4. Capability Score Distributions For Binary Items (Overall Mean & 95% CI) with Stratified Adjusted Means & 95% CI

eTable 1. Comparison of Sample Frame and Study Population at Time of Initial Assignment (2015)

eTable 2. Composite Capability Score Items

eTable 3. Practice Characteristics by Survey Year Stratified by Accountable Care Organization Participation

eTable 4. Practice Characteristics by Survey Year, Stratified by Ownership

eTable 5. Unweighted Median and Interquartile Ranges for Select Capability Scores

eTable 6. Detailed Comparisons of Primary Care Practice Capabilities, Stratified by ACO Participation

eTable 7. Detailed Comparisons of Primary Care Practice Capabilities, Stratified by Ownership

eTable 8. Comparison of Average Capability Score by ACO Participation and Ownership from Multi-level, Mixed-effects Model

eTable 9. Distribution of Average Capability Scores Over Time, in Total and Stratified by ACO Payment Participation and Practice Ownership

This supplemental material has been provided by the authors to give readers additional information about their work.

© 2025 Mackwood M et al. *JAMA Health Forum*.

eMethods

Additional Detail on Survey and Analysis Methods

The National Survey of Healthcare Organizations and Systems second wave (NSHOS Wave 2) was a 52- item questionnaire with 7 sections: background information; patient care; care of patients with complex needs and a high level of need; patient-reported measures, motivational interviewing and shared decision making; information systems and performance; COVID-19 response; and practice finances and payment. Intended to survey primary care practice leaders (e.g., practice managers or physician leaders), the development of the survey items and relevant response categories was based on extensive semi-structured interviews and focus groups with primary care physicians, practice leaders, and study consultants, dating back to 2000 when the first National Study of Physician Organizations (NSPO) survey was launched; this survey later evolved into the 2017-18 NSHOS. NSHOS 2017-2018 sampling included a range of practice sizes and system types and surveys at the primary care practice level, hospital level, and system level. NSHOS 2022-23 sampling included all NSHOS 2017-18 primary care practice respondents to maximize the potential to draw longitudinal inferences.

To provide further detail on sampling for NSHOS 2017-18: The sampling probabilities for the 2017-2018 survey corresponded to the use of a stratified-cluster sampling design to draw samples of organizations (systems, hospitals and physician practices) according to their complexity (whether there were multiple layers of ownership and the size of the organization). The rationale for this design was that even in 2017 the landscape of health systems exhibited extreme heterogeneity. Furthermore, in order to ensure that a greater number of sampled systems had responses from their hospitals and practices, the sampling designs for the ownership, hospital and practice surveys were coupled so that if any component of a system was sampled the owner and at least some of the other practices and hospitals within that system were sampled. The Monte Carlo algorithm abided to the following constraints: (1) for every sampled system, multiple organizations (subsidiaries, hospitals, practices) will also be sampled; (2) the probability of sampling an organization (system or medical group) will increase with the number of hospitals or medical practices within it; (3) subsidiary integrated delivery systems are included only if their parent is sampled and if a complex system owns one or more subsidiary integrated systems, then at least one of these will be sampled; (4) if any system is sampled and has 4 or fewer practices or hospitals, all of these will be sampled; (5) if any system has 4 or more practices or hospitals, the probability of sampling will increase logarithmically from a minimum of 4 to a maximum of 10. To select the sample and calculate sampling probabilities for each organization that adhere to these constraints, we used a Monte-Carlo algorithm to solve the implied system of mathematical equations, in turn enabling us to draw population-representative inferences; O'Malley and Park (2020) contains a detail description of the sampling design of the original NSHOS[1].

NSHOS 2022-2023 recruitment occurred as described in the manuscript, employing SSRS with a pre-specified protocol for scripted outreach to up to three contacts at each identified organization until response was obtained. Surveys were administered from June 2022 – February 2023 time period. All recorded data were encoded and securely stored with unique, de-identified IDs.

© 2025 Mackwood M et al. *JAMA Health Forum*.

Surveys which were > 50% incomplete were excluded. To account for non-response and loss to follow up, reported responses were weighted based upon the marginal probability of a practice responding to both 2017 and 2022 surveys. Because the longitudinal comparisons require that a practice return both surveys, all analyses in this paper were performed using survey weights to account for the marginal probability that a practice was 1) sampled from the original 2017 sampling frame, 2) responded to the 2017-2018 NSHOS survey, and 3) responded to the 2022 NSHOS survey. The probability of non-response to the 2017 survey was estimated using a logistic regression of the binary indicator of whether the practice was a non-respondent to the survey regressed on the variables that comprised the sampling design. In contrast, the probability of non-response in 2022 was simply estimated by the proportion of practices that did not respond. The marginal probability of being sampled and responding to both surveys was the product of the sampling probability, one minus the non-response probability to the wave 1 NSHOS, and one minus the non-response probability to the wave 2 survey. The marginal weight used in our statistical analyses was the inverse of the product of these three probabilities. The use of these weights allows the sample used for our analyses to match the entire sampling-frame for the original 2017-2018 survey.

eFigure 1 is a respondent flow chart detailing the numbers for practices ultimately included in analysis; **eTable 1** describes final analytic sample alongside the characteristics of the sample frame.

The composite scores reported in the main analyses are summary scores (linear combinations of individual items) calculated for each of the 9 practice scales (see etable 2 for component items). Because there were various missing data patterns across the survey items comprising each scale, we standardized each item by its sample-wide mean and standard deviation prior to averaging the resulting values, yielding standardized scales.

1. O'Malley AJ, Park S. A novel cluster sampling design that couples multiple surveys to support multiple inferential objectives. *Health Serv Outcomes Res Methodol*. 2020;20(2-3):85-110. doi:10.1007/S10742-020-00210-Y/FIGURES/5

© 2025 Mackwood M et al. *JAMA Health Forum*.

eFigure 1. Respondent Flow Chart

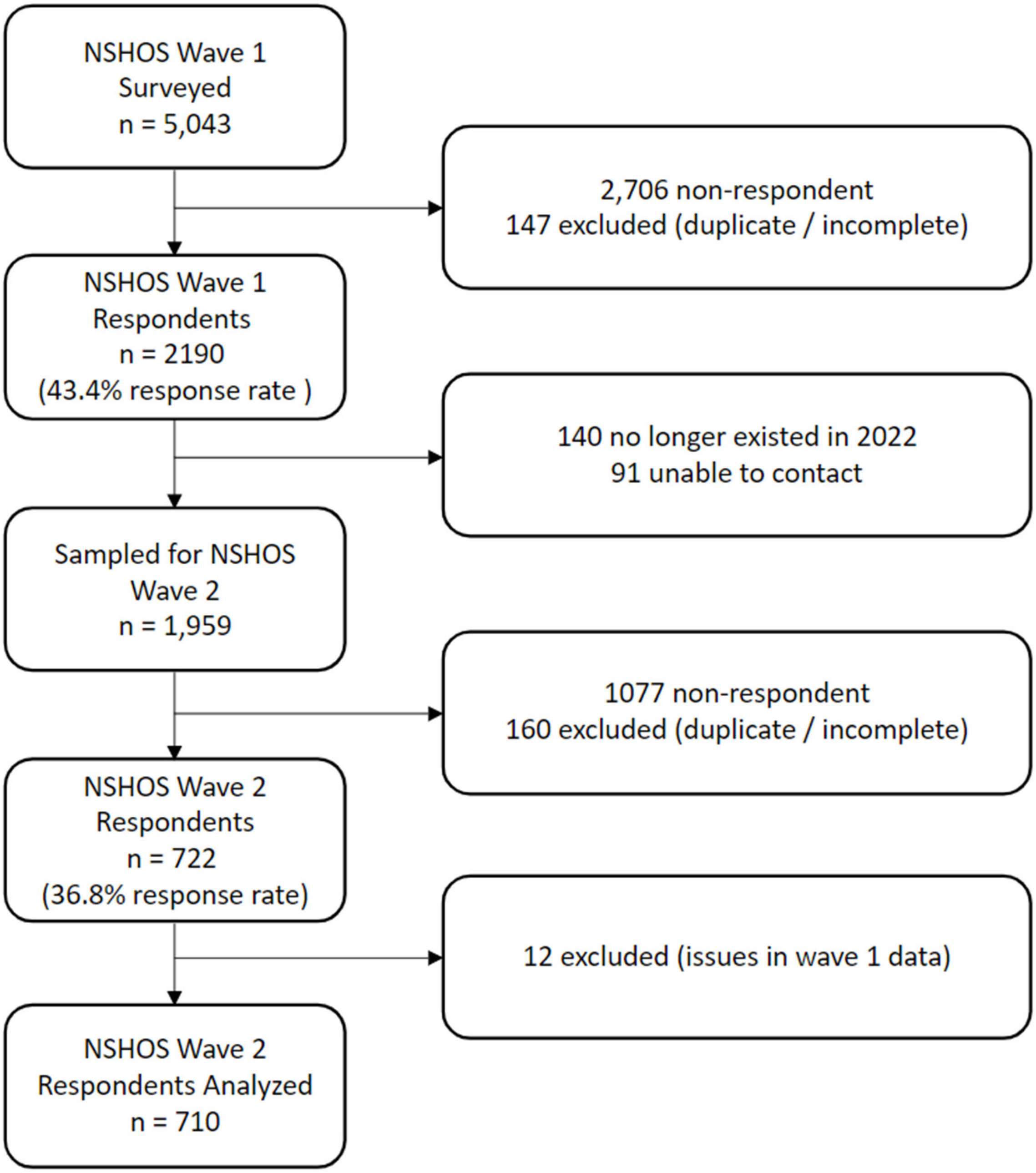

© 2025 Mackwood M et al. *JAMA Health Forum*.

eFigure 2. Average & Select Capability Score Distributions (Median & IQR), with Stratified Adjusted Means & 95% CI

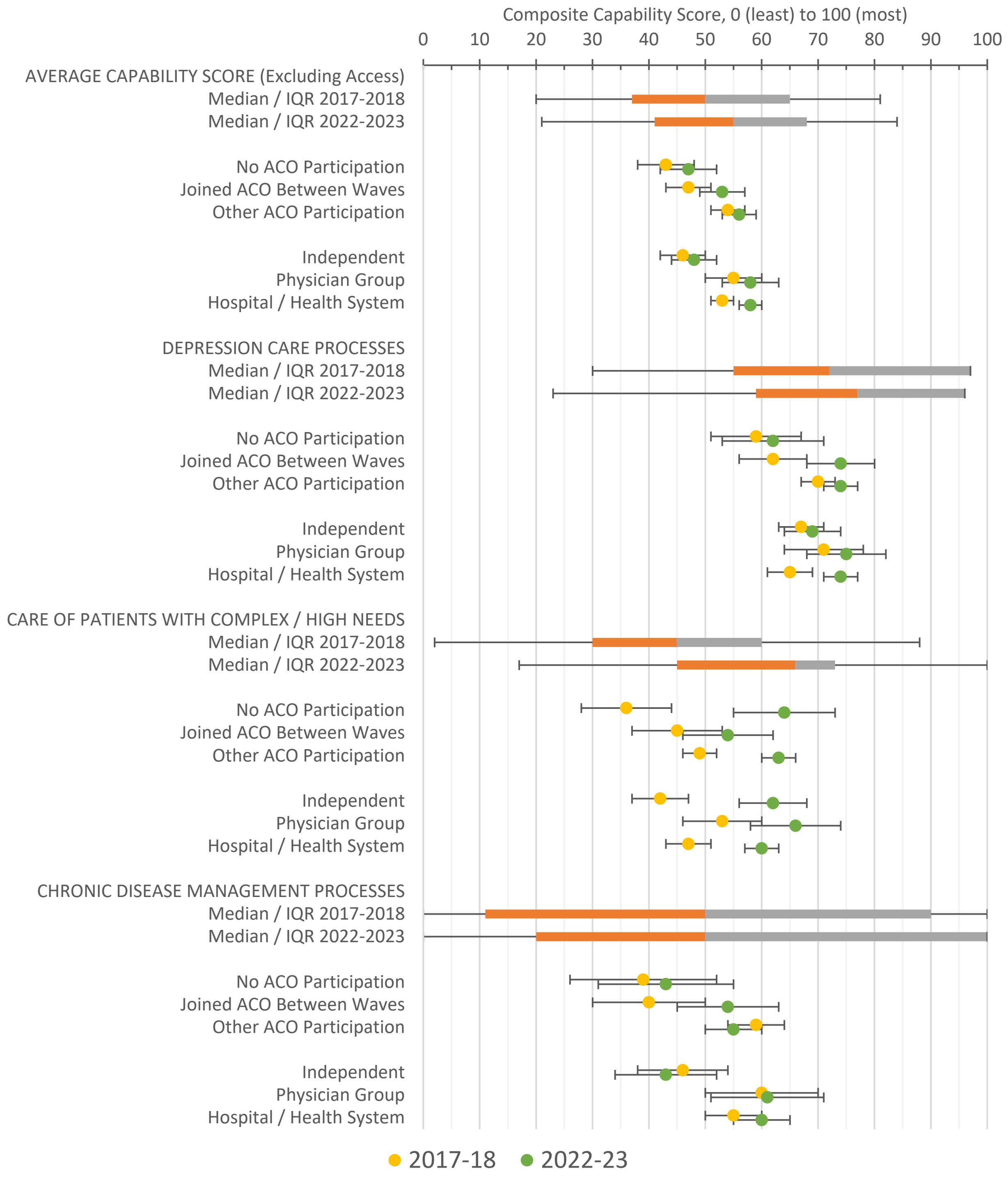

IQR = Interquartile Range. CI = Confidence Interval. Adjusted for ACO participation and ownership.
For median / IQR figures, central line denotes the median; boxes represent the interquartile range; whiskers display 95th percentile. For means, whiskers display 95% confidence interval

© 2025 Mackwood M et al. *JAMA Health Forum*.

eFigure 3. Additional Capability Score Distributions (Median & IQR), with Stratified Adjusted Means & 95% CI

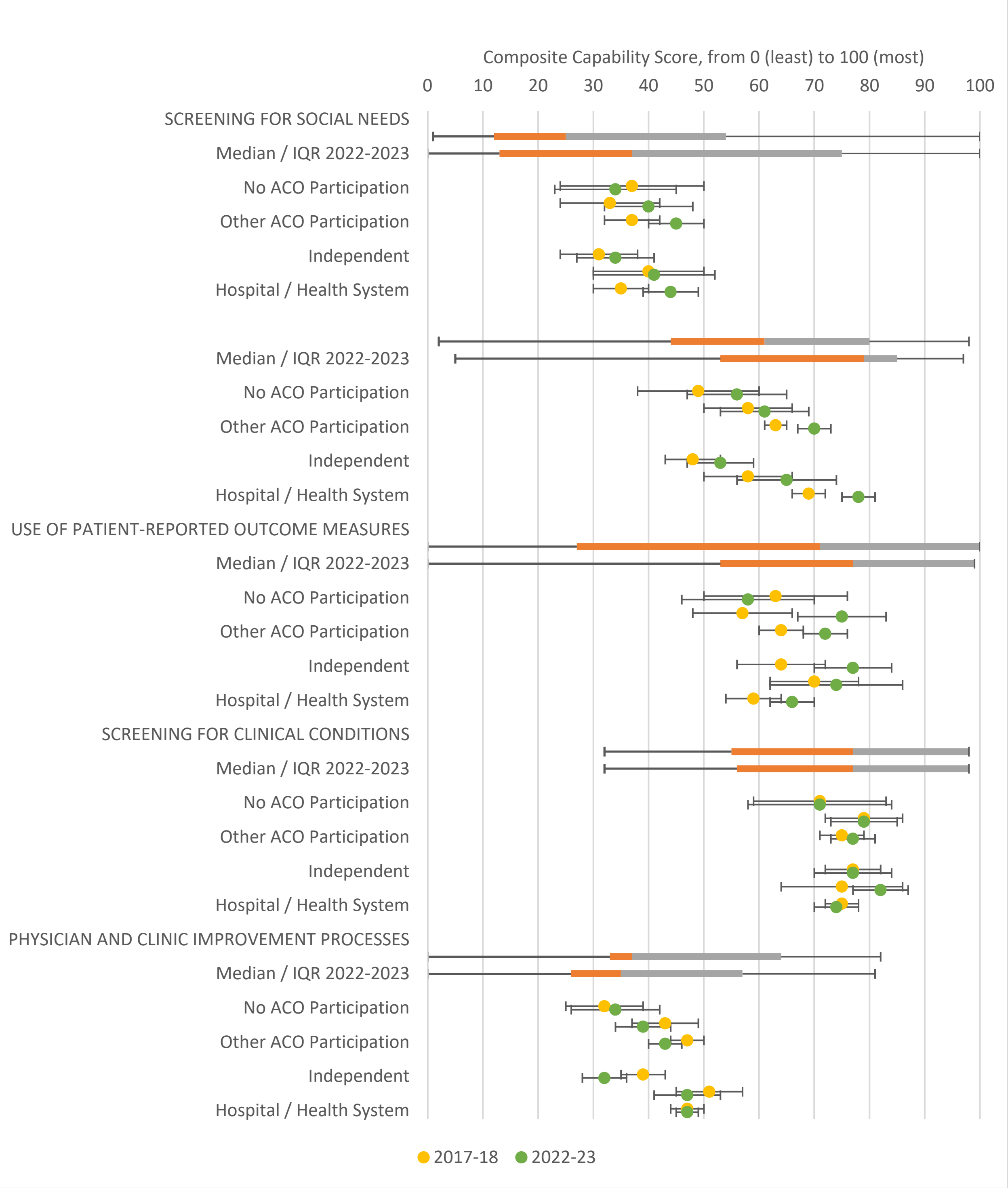

IQR = Interquartile Range. CI = Confidence Interval. Adjusted for ACO participation and ownership.
For median / IQR figures, central line denotes the median; boxes represent the interquartile range; whiskers display 95th percentile. For means, whiskers display 95% confidence interval

© 2025 Mackwood M et al. *JAMA Health Forum*.

eFigure 4. Capability Score Distributions For Binary Items (Overall Mean & 95% CI) with Stratified Adjusted Means & 95% CI

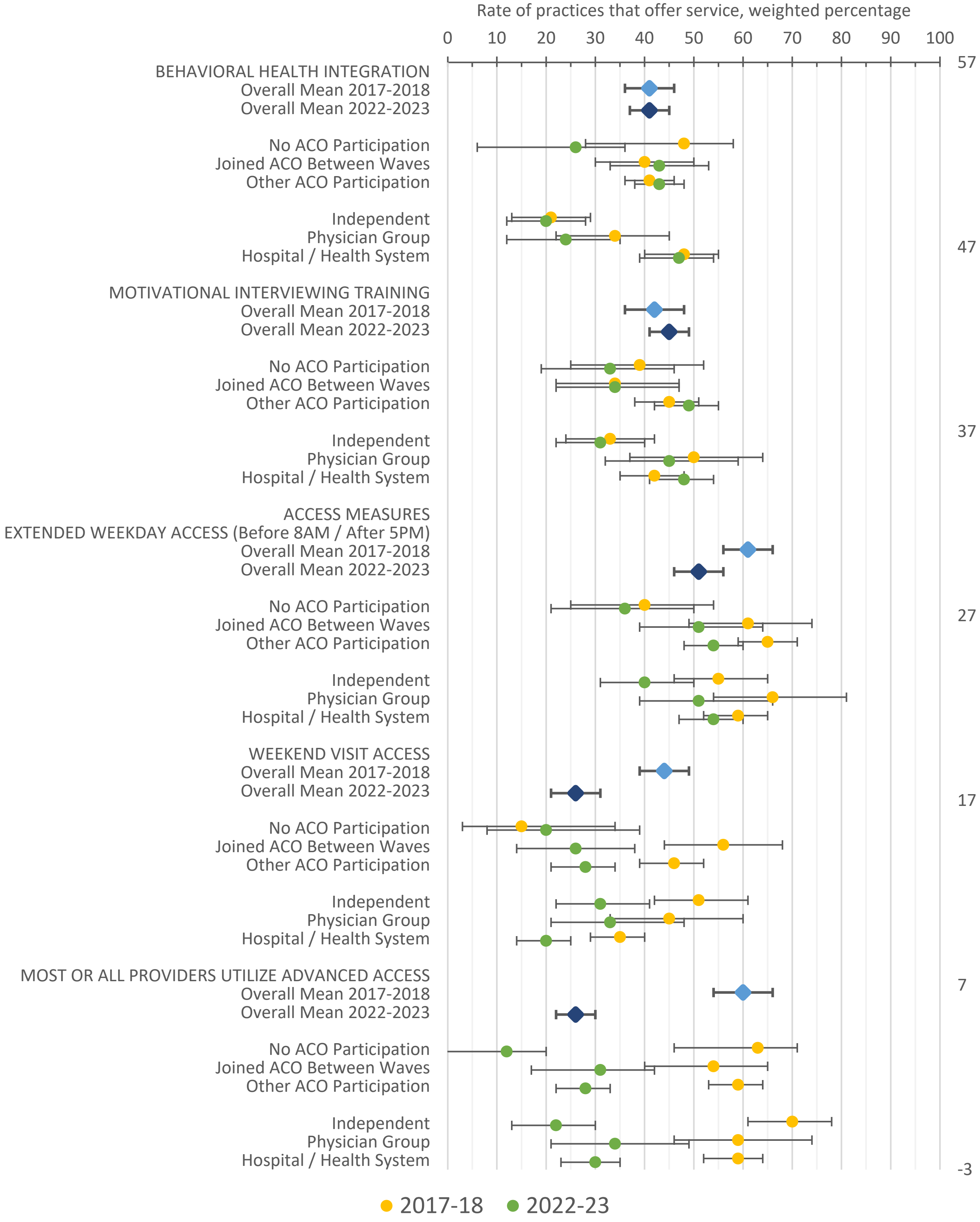

© 2025 Mackwood M et al. *JAMA Health Forum*.

eTable 1. Comparison of Sample Frame and Study Population at Time of Initial Assignment (2015)

Characteristic	Sample Frame	Longitudinal Respondents
	N = 15,768	N = 710
Ownership		
Independent or Physician Group Ownership	62.4% (9833)	50.6% (359)
Hospital or Health System Ownership	37.5% (5920)	49.4% (351)
Physician Count		
Small(0-4)	41.7% (6573)	40.6% (288)
Medium(5-9)	36.2% (5705)	37.2% (264)
Large(10-19)	13.5% (2126)	13.0% (92)
Very Large (20+)	8.6% (1360)	9.3% (66)
Rurality		
Not rural	93.1% (14684)	91.7% (651)
Rural	6.8% (1079)	8.2% (58)
Census Region		
New England	6.2% (981)	8.6% (61)
Middle Atlantic	14.2% (2242)	11.8% (84)
East North Central	17.6% (2783)	15.2% (108)
West North Central	8.3% (1314)	11.4% (81)
South Atlantic	18.5% (2912)	16.9% (120)
East South Central	4.8% (759)	3.2% (23)
West South Central	8.7% (1378)	6.9% (49)
Mountain	7.0% (1109)	9.9% (70)
Pacific	14.5% (2287)	16.1% (114)

© 2025 Mackwood M et al. *JAMA Health Forum*.

eTable 2. Composite Capability Score Items

Composite item & component questions	Cronbach's Alpha	
	**2017-18**	**2022-23**
Average Capability Composite<br>*All below unique individual items (k = 41)*	0.90	0.90
Behavioral Health Integration<br>*Do you have behavioral health services formally integrated into your practice? (yes/no)*	n/a	n/a
Motivational Interviewing Training<br>*After a brief description of motivational interviewing:*<br>*Does your practice have clinicians/staff who are formally trained in motivational interviewing? (yes / no)*	n/a	n/a
Depression Care Processes<br>*Does your practice have a system in place to routinely screen patients for depression? (yes/no)*<br>*Does your practice collect patient-reported measures of depression? (yes/no)*<br>*Does your practice currently use any EHR-based clinical decision-support tools (e.g. embedded order sets) to improve adherence to evidence-based care for depression? (yes/no)*<br>*Does your practice maintain a list or registry to help manage the care of patients with depression? (yes/no)*	0.51	0.47
Screening for Social Needs<br>*Does your practice have a system in place to routinely screen patients for (yes/no):*<br>• *Low health literacy*<br>• *Food insecurity*<br>• *Housing instability*<br>• *Utility needs*<br>• *Interpersonal violence*<br>• *Transportation needs*<br>• *Need for financial assistance with medical bills*<br>• *Medicaid eligibility*	0.86	0.89
Care Processes for Patients with Complex Needs and a High Level of Need<br>*For your complex, high need patients, how often is a non-physician in the practice involved in: (never / sometimes / often / always):*<br>• *Helping the patient coordinate care across clinicians*<br>• *Helping the patient adhere to the care plan*<br>• *Supporting health risk modification*<br>• *Supporting medication adherence*	0.84	0.90

© 2025 Mackwood M et al. *JAMA Health Forum*.

Composite item & component questions	Cronbach's Alpha 2017-18	Cronbach's Alpha 2022-23
Electronic Health Record (EHR) Integration<br>*Does your practice's EHR connect directly to the EHR at the main hospital that your patients use? (Yes, single EHR; Yes, different EHR, but one that is fully interoperable; Yes, different EHR, but partially interoperable; No, not connected)*<br>*Does your practice's health information system (including your EHR) allow (yes/no):*<br>• *Patients to have electronic access to their medical records*<br>• *Patients to electronically comment on and/or input information to their medical records (such as Open Notes)*<br>• *Physicians and patients to communicate with one another via email*<br>• *Physicians to know whether their patients have filled their prescriptions*	0.59	0.64
Patient-Reported Outcome Measures<br>*Does your practice collect patient-reported measures of depression? (yes/no)*<br>*Does your practice collect patient-reported measures of physical function or disability for older adult patients? (yes/no)*<br>*Does your practice collect patient-reported measures of pain for diabetic patients? (yes/no)*<br>*Does your practice collect patient-reported measures of pain for musculoskeletal hip, knee or back patients? (yes/no)*	0.75	0.77
Screening for Clinical Conditions<br>*Does your practice have a system in place to routinely screen patients for (yes/no):*<br>• *Tobacco use*<br>• *Opioid use specifically*<br>• *Substance use disorders (other than tobacco and opioid)*<br>• *Polypharmacy*	0.63	0.62
Physician & Clinic Improvement Processes<br>*How does your practice use information about individual clinician performance for (check any: we don't use it / use for feedback / use for internal quality improvement / use for physician compensation):*<br>• *Preventive services (e.g. immunizations, screening)*<br>• *Patient experiences (e.g. patient satisfaction or CAHPS scores)*<br>• *Clinical quality (e.g. blood pressure control, diabetes control, complication rates)*<br>• *Overuse of medical tests or procedures (e.g. high cost imaging)*	0.74	0.74

© 2025 Mackwood M et al. *JAMA Health Forum*.

Composite item & component questions	Cronbach's Alpha 2017-18	Cronbach's Alpha 2022-23
Chronic Disease Management Processes<br>*Does your practice currently use any EHR-based clinical decision-support tools (e.g. embedded order sets) to improve adherence to evidence-based care for (yes/no):*<br>• *Diabetes*<br>• *Congestive heart failure*<br>• *Asthma / COPD*<br>• *Hypertension*<br>• *Depression*<br>*Does your practice maintain a list or registry to help manage the care of patients with (yes/no):*<br>• *Diabetes*<br>• *Congestive heart failure*<br>• *Asthma / COPD*<br>• *Hypertension*<br>• *Depression*	0.91	0.92
**Non-composite items**		
Background Information<br>*Is your practice: (A Federally Qualified Health Center (FQHC) / A FQHC "look-alike" (do not select unless you have this designation) / Not designated as either / Don't know)*<br>*Who owns your practice? (Independently owned / A larger physician group / A hospital / A healthcare system (may include a hospital) / Other)*<br>*How many primary care physicians (MD/DO) work at your practice location? (Insert #)*	n/a	n/a
Access to care<br>*Is your practice regularly open for patient appointments on… (do not include urgent care provided in a different location) (yes/no)*<br>• *Weekdays before 8am or after 5pm?*<br>• *Weekends?*<br>*How many physicians in your practice use the 'advanced access' or 'open access' schedule method on a regular basis to offer same-day appointments? (Not familiar with 'advanced access' or 'open access' scheduling / None / Some / Most / All)*	n/a	n/a

© 2025 Mackwood M et al. *JAMA Health Forum*.

Composite item & component questions	Cronbach's Alpha 2017-18	2022-23
Payment / ACO participation<br>*Please rate how your practice is doing financially now. (Poor / Fair / Good / Very good)*<br>*Has your practice ever participated in any of these payment and delivery reform initiatives? (Yes, currently / Yes, previously but not now / No, never):*<br>• *Capitated contracts with commercial health plans*<br>• *Medicare ACO contracts*<br>• *Commercial ACO contracts*<br>• *Medicaid ACO contracts*	n/a	n/a

© 2025 Mackwood M et al. *JAMA Health Forum*.

eTable 3. Practice Characteristics by Survey Year Stratified by Accountable Care Organization Participation

	No ACO Participation			Joined Between Surveys			ACO Participation (Excluding Joiners)		
Domain / item - reported as weighted percentage (unweighted count)	2017-18	2022 - 23	p value for difference between years (chi-squared)	2017-18	2022 - 23	p value for difference between years (chi-squared)	2017-18	2022 - 23	p value for difference between years (chi-squared)
**Ownership**									
Independent	60 (30)	53 (27)	0.12	39 (34)	36 (28)	0.19	33 (156)	27 (127)	0.00
Physician group	2 (3)	1 (2)		17 (20)	16 (20)		15 (76)	11 (58)	
Hospital or Health System	30 (31)	38 (35)		37 (46)	41 (52)		42 (222)	52 (267)	
FQHC	8 (4)	8 (4)		7 (7)	7 (7)		10 (32)	10 (34)	
**Changes in Ownership**									
Group to Hospital/System	1 (1)			3 (5)			7 (31)		
Hospital to System	4 (5)			8 (8)			8 (33)		
Independent to FQHC	0			0			0.2 (2)		
Independent to Group	0			3 (5)			3 (13)		
Independent to Hospital/System	7 (3)			1 (1)			3 (14)		
No Change	89 (59)			86 (88)			80 (393)		
**Physician Count**									
0-4	59 (32)	62 (36)	0.55	51 (46)	42 (41)	0.25	42 (179)	46 (187)	0.30
5-9	15 (18)	13 (15)		20 (30)	26 (32)		34 (165)	31 (154)	

© 2025 Mackwood M et al. *JAMA Health Forum*.

Domain / item - reported as weighted percentage (unweighted count)	No ACO Participation 2017-18	No ACO Participation 2022 - 23	No ACO Participation p value for difference between years (chi-squared)	Joined Between Surveys 2017-18	Joined Between Surveys 2022 - 23	Joined Between Surveys p value for difference between years (chi-squared)	ACO Participation (Excluding Joiners) 2017-18	ACO Participation (Excluding Joiners) 2022 - 23	ACO Participation (Excluding Joiners) p value for difference between years (chi-squared)
10-19	19 (11)	18 (9)		16 (15)	19 (17)		13 (77)	12 (75)	
20+	7 (7)	7 (8)		13 (16)	13 (17)		11 (65)	10 (64)	
**Advanced Practice Provider (APRN, PA) Count**									
Zero	30 (18)	25 (15)	0.44	20 (25)	15 (20)	0.50	18 (82)	18 (83)	0.07
1 or 2	40 (28)	38 (30)		30 (35)	30 (33)		36 (191)	31 (161)	
3 or 4	24 (12)	24 (12)		29 (26)	31 (31)		22 (104)	21 (103)	
5 to 10	5 (7)	10 (8)		15 (14)	14 (14)		17 (69)	22 (83)	
10+	2 (3)	2 (3)		5 (7)	10 (9)		7 (40)	9 (50)	
**Ratio of Advanced Practice Providers to Physicians**									
Less than 0.5	60 (48)	62 (42)	0.66	58 (68)	53 (60)	0.04	60 (325)	51 (273)	0.00
0.5 to less than 1	32 (15)	25 (16)		23 (24)	15 (18)		20 (91)	23 (99)	
1 to less than 2	8 (5)	10 (7)		18 (14)	18 (21)		18 (59)	14 (82)	
2+	0 (0)	4 (2)		1 (1)	13 (8)		3 (11)	13 (25)	
Geographic Characteristics									

© 2025 Mackwood M et al. *JAMA Health Forum*.

Domain / item - reported as weighted percentage (unweighted count)	No ACO Participation			Joined Between Surveys			ACO Participation (Excluding Joiners)		
	2017-18	2022 - 23	p value for difference between years (chi-squared)	2017-18	2022 - 23	p value for difference between years (chi-squared)	2017-18	2022 - 23	p value for difference between years (chi-squared)
**Practice in a Rural Location***	10 (7)			12 (15)			4 (29)		
**US census region**									
New England	1 (1)			4 (3)			10 (53)		
Middle Atlantic	8 (6)			23 (18)			13 (54)		
East North Central	7 (7)			12 (17)			14 (75)		
West North Central	8 (9)			12 (12)			12 (56)		
South Atlantic	23 (19)			17 (16)			17 (78)		
East South Central	4 (1)			4 (7)			3 (12)		
West South Central	15 (8)			9 (11)			5 (27)		
Mountain	6 (5)			6 (7)			11 (53)		
Pacific	29 (12)			12 (16)			17 (78)		

[a]Commercial, Medicare, Medicaid
[b]Determined by Rural-Urban Commuting Area Classification for the practice's business address

© 2025 Mackwood M et al. *JAMA Health Forum*.

eTable 4. Practice Characteristics by Survey Year, Stratified by Ownership

Domain / item - reported as weighted percentage (unweighted count)	Independent Ownership			Physician Group Ownership			Hospital or System Ownership		
	2017-18	2022 - 23	p value for difference between years (chi-squared)	2017-18	2022 - 23	p value for difference between years (chi-squared)	2017-18	2022 - 23	p value for difference between years (chi-squared)
**n**	234	193		105	85		321	380	
**Accountable Care Organization Participation**									
No ACO participation (at either time)	24 (30)	25 (27)	0.40	2 (3)	2 (2)	0.43	12 (31)	12 (35)	0.30
Joined ACO between surveys	16 (34)	17 (28)		20 (20)	25 (20)		14 (46)	13 (52)	
ACO participation	60 (156)	58 (127)		78 (76)	74 (58)		74 (222)	75 (267)	
**Accountable Care Organization Participation**									
Mean No. of ACO by Payer Type[a] - mean (sd)	1.0 (0.11)	1.4 (0.15)	<0.01	1.4 (0.13)	2.0 (0.13)	<0.01	1.5 (0.09)	1.7 (0.09)	0.07
Zero ACO Payer Types	34 (73)	21 (38)	<0.01	28 (23)	14 (11)	<0.01	30 (83)	27 (92)	0.06
One ACO Payer Type	32 (69)	22 (40)		18 (15)	16 (13)		19 (53)	11 (36)	
Two ACO Payer Types	19 (42)	33 (60)		34 (28)	33 (26)		20 (54)	16 (56)	
Three ACO Payer Types	15 (33)	25 (45)		20 (17)	38 (30)		31 (86)	46 (156)	
**Physician Count**									
0-4	62 (130)	65 (114)	0.24	36 (38)	49 (38)	0.06	37 (103)	36 (120)	0.53
5-9	24 (70)	20 (53)		36 (35)	22 (22)		30 (105)	33 (126)	
10-19	9 (18)	11 (16)		14 (17)	16 (15)		17 (56)	15 (58)	
20+	5 (16)	4 (10)		14 (15)	13 (10)		15 (57)	16 (71)	
**Advanced Practice Provider (APRN, PA) Count**									
Zero	24 (54)	26 (48)	0.04	14 (14)	16 (11)	0.95	22 (62)	17 (65)	0.049
1 or 2	33 (89)	30 (63)		38 (41)	36 (31)		42 (136)	35 (138)	

© 2025 Mackwood M et al. *JAMA Health Forum*.

Domain / item - reported as weighted percentage (unweighted count)	Independent Ownership			Physician Group Ownership			Hospital or System Ownership		
	2017-18	2022 - 23	p value for difference between years (chi-squared)	2017-18	2022 - 23	p value for difference between years (chi-squared)	2017-18	2022 - 23	p value for difference between years (chi-squared)
3 or 4	28 (51)	20 (37)		27 (25)	24 (22)		20 (64)	26 (87)	
5 to 10	11 (27)	20 (30)		16 (19)	20 (17)		9 (34)	15 (52)	
10+	4 (13)	4 (15)		5 (6)	4 (4)		7 (25)	7 (33)	
**Ratio of Advanced Practice Providers to Physicians**									
Less than 0.5	54 (144)	52 (98)	0.04	65 (70)	52 (45)	0.41	72 (240)	61 (238)	<0.01
0.5 to less than 1	26 (51)	20 (37)		22 (21)	20 (20)		17 (51)	22 (75)	
1 to less than 2	19 (36)	17 (42)		7 (10)	14 (15)		11 (27)	13 (51)	
2+	2 (3)	11 (15)		5 (4)	14 (4)		0 (3)	4 (10)	
Geographic Characteristics									
**Practice in a Rural Location**[b]	0 (4)			7 (8)			11 (40)		
**US census region**									
New England	7 (10)			2 (3)			9 (42)		
Middle Atlantic	17 (29)			14 (10)			13 (41)		
East North Central	24 (21)			16 (12)			22 (69)		
West North Central	41 (19)			30 (9)			35 (51)		
South Atlantic	65 (45)			47 (21)			56 (47)		
East South Central	107 (10)			77 (2)			91 (8)		
West South Central	172 (19)			123 (8)			147 (18)		
Mountain	278 (14)			200 (8)			238 (39)		
Pacific	450 (26)			323 (12)			385 (65)		

[a]Commercial, Medicare, Medicaid
[b]Determined by Rural-Urban Commuting Area Classification for the practice's business address

© 2025 Mackwood M et al. *JAMA Health Forum*.

eTable 5. Unweighted Median and Interquartile Ranges for Select Capability Scores[a]

	Survey years	
**Practice Capability Composite Scores** (Median (IQR))	**17-18**	**22-23**
Average Capability Score (excluding access)	52 (40 - 66)	56 (43 - 70)
Depression Care Processes	76 (55 - 97)	78 (59 - 96)
Screening for Social Needs	25 (12 - 52)	37 (12 - 75)
Care of Patients with Complex / High Needs	46 (31 - 64)	66 (45 - 73)
Electronic Health Record Integration	63 (44 - 80)	79 (63 - 91)
Patient-Reported Outcome Measures	75 (27 - 100)	77 (53 - 99)
Screening for Clinical Conditions	77 (55 - 98)	77 (56 - 98
Physician & Clinic Improvement Processes	41 (33 - 67)	35 (26 - 59)
Chronic Disease Management Processes	50 (20 - 99)	50 (21 - 100)

[a]Access scores, as well as behavioral health integration and motivational interviewing training, are single-item responses and not included.

© 2025 Mackwood M et al. *JAMA Health Forum*.

eTable 6. Detailed Comparisons of Primary Care Practice Capabilities, Stratified by ACO Participation

	No ACO Participation (Referent)		Joined ACO Between Surveys		ACO Participation (excluding Joiners)	
	**2017-18**	**2022-23**	**2017-18**	**2022-23**	**2017-18**	**2022-23**
**Practice Capability Composite Scores (Mean, 100-point scale)**	n = 68		n = 107		n = 486	
Average of all included capabilities (excluding access)	41	45	47	53*	54***	57***
Behavioral Health Integration	41	23	36	40	40	45**
Motivational Interviewing Training	35	31	33	33	45	50*
Depression Care Processes	59	61	62	74*	70**	75**
Screening for Social Needs	34	32	33	39	38	46*
Care of Patients with Complex / High Needs	34	64	46*	55	49***	63
Electronic Health Records Integration	45	53	57	63	63**	72**
Patient Reported Outcome Measures	63	60	58	74	64	72
Screening for Clinical Conditions	71	71	79	80	76	77
Physician & Clinic Improvement Processes	29	32	43**	39	47***	44**
Chronic Disease Management Processes	37	40	40	54	59**	57*
**Reported Care Access (Weighted percentage of respondents)**						
Offer Extended Hours[a]	38	34	61*	50	65**	55*
Offer Weekend Visits	18	22	57***	27	47***	27
Utilize Open Access Scheduling (most or all providers)	65	11	54	34**	59	29**

[a]Visits before 8am or after 5pm on weekdays

For difference within a time period compared to referent category: * $p < 0.05$, ** $p < 0.01$, *** $p < 0.001$

Comparisons calculated across groups with univariate ordinal least squares regression

© 2025 Mackwood M et al. *JAMA Health Forum*.

eTable 7. Detailed Comparisons of Primary Care Practice Capabilities, Stratified by Ownership

	Independent Ownership (Referent)		Physician Group Ownership		Hospital or System Ownership	
	2017-18	2022-23	2017-18	2022-23	2017-18	2022-23
**Practice Capability Composite Scores (Mean, 100-point scale)**	n = 234	n = 193	n = 105	n= 85	n = 321	n = 380
Average of all included capabilities (excluding access)	45	47	56***	59***	53***	58***
Behavioral Health Integration	22	19	33	26	48***	48***
Motivational Interviewing Training	33	30	51*	43	41	49**
Depression Care Processes	66	68	72	76	65	74*
Screening for Social Needs	31	33	39	42	36	44*
Care of Patients with Complex / High Needs	41	62	55**	65	47	60
Electronic Health Records Integration	47	53	60**	67*	70***	79***
Patient Reported Outcome Measures	65	76	70	80	59	66*
Screening for Clinical Conditions	77	76	75	83	75	74
Physician & Clinic Improvement Processes	38	31	53***	48***	48**	47***
Chronic Disease Management Processes	45	42	62**	63**	56*	61***
**Reported Care Access (Weighted percentage of respondents)**						
Offer Extended Hours[a]	53	38	69**	53	60	54*
Offer Weekend Visits	48	30	51	34	36	21
Utilize Open Access Scheduling (most or all providers)	69	23	58	38	60	30

[a]Visits before 8am or after 5pm on weekdays

For difference within a time period compared to referent category: * $p < 0.05$, ** $p < 0.01$, *** $p < 0.001$

Comparisons calculated across groups with univariate ordinal least squares regression

© 2025 Mackwood M et al. *JAMA Health Forum*.

eTable 8. Comparison of Average Capability Score by ACO Participation and Ownership from Multi-level, Mixed-effects Model[a]

	Model Estimated Average Capability Score	95% CI
Model Constant	46	41 to 51
Time period 2 (vs time period 1)	3.8	-2.7 to 10
ACO participation (vs non)	4.6	-0.15 to 9.3
ACO participation * time interaction	0.11	-6.1 to 6.3
Group ownership (vs independent)	5.6	-2.0 to 13
Group ownership * time interaction	-2.5	-9.7 to 4.7
Hospital / Health System ownership (vs independent)	-0.0038	-7.1 to 6.3
Hospital / Health System * time interaction	1.4	-4.3 to 7.1
**Variation Constants**		
System Clusters	2.0	1.7 to 2.4
Overall	1.5	1.3 to 1.7
**Change in Average Capability Score Over Time**		
No ACO participation	3.7	-1.2 to 8.7
ACO participation	3.9*	0.49 to 7.2
Independent Ownership	3.8	-0.86 to 8.4
Group Ownership	1.3	-4.2 to 6.7
Hospital / Health System Ownership	5.2**	1.8 to 8.5

* p < 0.05; ** p < 0.01

[a]Generalized linear mixed-effects model, with multiple levels for system and practice (nested within systems as applicable) random effects and indicators for time, ownership and ACO participation

© 2025 Mackwood M et al. *JAMA Health Forum*.

eTable 9. Distribution of Average Capability Scores Over Time, in Total and Stratified by ACO Payment Participation and Practice Ownership

Category	Survey Years	n	Median	Interquartile Range
**Overall**	2017 - 18	710	50	37 to 65
	2022 - 23	707	55	41 to 68
**Accountable Care Organization (ACO) Participation**				
**No ACO Participation**	2017 - 18	68	34	24 to 56
	2022 - 23	67	47	34 to 58
**Joined ACO Between Waves**	2017 - 18	107	41	32 to 61
	2022 - 23	107	56	44 to 62
**Other ACO Participation**	2017 - 18	486	53	42 to 66
	2022 - 23	485	57	43 to 72
**Ownership**				
**Independent**	2017 - 18	234	43	28 to 58
	2022 - 23	192	47	33 to 58
**Physician Group**	2017 - 18	105	58	39 to 75
	2022 - 23	85	63	44 to 75
**Hospital / Health System**	2017 - 18	321	52	43 to 66
	2022 - 23	379	59	48 to 70

© 2025 Mackwood M et al. *JAMA Health Forum*.
