# Supplementary material for: Changes in US Primary Care Access and Capabilities During the COVID-19 Pandemic
Source: JAMA Health Forum. 2025 Feb 7;6(2):e245237. doi: 10.1001/jamahealthforum.2024.5237 (PMC11806387; doi:10.1001/jamahealthforum.2024.5237)
Supplement: Supplement 2. — Data Sharing Statement [file jamahealthforum-e245237-s002.pdf]

Data Sharing Statement

Mackwood. Changes in US Primary Care Access and Capabilities During the COVID-19 Pandemic. *JAMA Health Forum*. Published February 07, 2025.
doi:10.1001/jamahealthforum.2024.5237

Data
Data available: Yes
Data types: Data (not involving human participants)
How to access data: karen.e.schifferdecker@dartmouth.edu
When available: With publication

Supporting Documents
Document types: Other (please specify)
Additional Information: Survey instrument
How to access documents: karen.e.schifferdecker@dartmouth.edu
When available: With publication

Additional Information
Who can access the data: researchers whose proposed use of the data has been approved
Types of analyses: for any purpose
Mechanisms of data availability: after approval of a proposal
